# The impact of the baby friendly hospital initiative on healthcare utilization among newborns insured by Medicaid in Delaware

**DOI:** 10.1186/s12887-023-04424-0

**Published:** 2023-12-04

**Authors:** Cecelia Harrison-Long, Mia Papas, David A. Paul

**Affiliations:** 1 Children’s Hospital of Philadelphia, Infection Prevention and Control, Philadelphia, USA; 2 Real World Evidence, AstraZeneca, Wilmington, DE USA; 3 Department of Pediatrics, Christiana Care Health System, 1M20, 4745 Ogletown Stanton Drive, Newark, DE 19718 USA

**Keywords:** Breast feeding, Readmission, Baby freindly hospital initiative

## Abstract

**Background:**

The Baby Friendly Hospital Initiative was created to enhance breastfeeding, although its impact on infant healthcare utilization is unclear. Breast feeding infants are vulnerable to readmission soon after birth secondary to dehydration and hyperbilirubinemia. Breastfeeding can also protect infants from unnecessary health care utilization later in life by preventing infection. The objective of this study was to examine the impact of the Baby Friendly Hospital Initiative on readmissions and emergency department utilization among Medicaid births in Delaware.

**Methods:**

The study was a quasi-experimental design. Medicaid claims files were used to study births at five hospitals in Delaware born between January 1, 2014, and December 31, 2018, and covered under Medicaid at time of birth. Three hospitals were designated Baby Friendly, two were not and served as controls. Outcomes included Emergency Department (ED) utilization and readmissions within 30 days and one-year of birth hospitalization. Exposure to the Baby Friendly Hospital Initiative was determined by year and hospital of birth. Logistic regression and interrupted time series segmented regression analysis with controls were used to assess the effect of Baby Friendly Hospital Initiative on healthcare utilization.

**Results:**

In total, 19,695 infants were born at five hospitals with 80% (15,939) born at hospitals that were designated Baby Friendly. ED utilization and readmissions over the 1st year of life for breastfeeding related diagnosis at the Baby Friendly hospitals occurred in 240 (1.5%) and 226 (1.4%) of infants, respectively. Exposure to the Baby Friendly Hospital Initiative was associated with increased odds of all cause 30-day readmission (AOR: 1.15; 95% CI: 1.03–1.28) but not readmissions over the 1st year of life. While 30-day ED visits did not change after BFHI, one-year ED visits were reduced (0.91, 95% CI 0.86–0.97). A significant negative trend was seen over time for ED utilization post BFHI compared to controls (B: -5.90, p < 0.01).

**Conclusion:**

There was a small observed increase in the odds of all cause 30-day readmissions with no change in one-year readmissions after BFHI in Delaware. Although there were no observed changes in 30-day ED utilization, there was a reduction in one-year ED utilization following the implementation of the Baby Friendly Hospital Initiative in Delaware birth hospitals. Our data help to inform policy and decision making for statewide systems of care that may be used to support breast feeding.

## Introduction

The medical benefits of breast feeding are clear and well established for both baby and mother. Despite the well-known advantages of providing mother’s milk, breast feeding rates differ by region, race, insurance status and socioeconomic status [1–4]. Baby Friendly designation and the accompanying Ten Steps to Successful Breast feeding has been used by multiple hospital systems to improve rates of breastfeeding [5, 6] despite some literature suggesting a lack of efficacy for this program in sustaining breastfeeding [7]. The Baby Friendly Initiative has the potential to improve breast feeding and overall health but has been reported to have some unintended consequences including readmission for hyperbilirubinemia [8] and may change outcomes for some babies by deemphasizing formula feeding and supplementation during the initial hospital stay [9]. While breast feeding will improve both long- and short-term health, exclusively breastfed infants are also at increased risk for excessive weight loss, dehydration, and hyperbilirubinemia in the immediate newborn period. Promoting breast feeding at the level of hospital systems thus has the potential to decrease unplanned healthcare utilization in the first year of life by reducing infection and improving other conditions associated with hospital admission and at the same time increase hospitalization early in life if breast feeding has not been well established.

The aims of this research were to investigate if the BFHI influences both short term (30 day) and longer term (one year) health care utilization at a state level. We hypothesized that BFHI would increase 30 day and reduce one-year readmissions and Emergency Department (ED) utilization. To meet these aims, hospital readmission and ED utilization were investigated in temporal association with initiation of Baby Friendly designation at birth hospitals in a single state. The study sample included a population of babies covered by Medicaid insurance in the State of Delaware.

## Methods

### Setting

We conducted a quasi-experimental study of births at all five birth hospitals in the state of Delaware between January 1, 2014, and December 31, 2018. All patients studied were covered under Medicaid at the time of birth. Approximately 45% of births were covered by Medicaid between 2014 and 2018 in Delaware. This study was approved by ChristianaCare and the State of Delaware Institutional Review Boards.

### Data collection

Data source for the study was the Medicaid administrative claim files. The study sample included infants having Medicaid as their sole payer born within the years 2014–2018. From these data, the following were recorded for the infants: sex, age in months, race, and year of birth. The birth facility for each infant was determined and infants were included in the study population if they were born at one of the five Delaware birth hospitals and were covered under Medicaid at time of birth, according to infants’ Medicaid claims. Infant diagnoses, preterm status, and healthcare utilization variables were obtained. Infants born out of the state of Delaware or who were not eligible for Medicaid during the first month of life were excluded. All data were provided by the University of Delaware Center for Community Research and Service who hold an agreement with the Delaware Division of Medicaid and Medical Assistance. A de-identified data set was provided to ChristianaCare under a Delaware INBRE Core access award for the current study. Data are not publicly available.

In order to investigate both short-term and longer-term health care utilization, we examined hospital readmissions and ED utilization both at 30 days and one year following initial hospital discharge. Thus, outcomes investigated included all cause 30-day readmission, all cause total readmissions within 1 year, all cause 30-day ED visits, and all cause total ED visits within 1 year. To compare frequency of utilization, we also analyzed the data based on a categorical stratification of zero, one, or two or more readmissions or ED visits. For the short term, 30-day analyses, we also investigated breast feeding related readmissions and breast-feeding related ED visits. Breast feeding related diagnoses included dehydration, hypernatremia, weight loss, failure to thrive, hyperbilirubinemia, and jaundice determined by ICD-9/10 coding. The date of birth of the infant was utilized as the baseline date to calculate 30-day and one-year outcomes.

### Data analysis

Three hospitals in the cohort were designated as Baby Friendly Hospitals (BFHI) during the study time-period and two were not designated as BFHI. The temporal clustering of hospital choosing to start the BFHI in 2016 was related to coordinated state-wide public health initiatives to increase breast feeding. The decision to ultimately implement the BFHI as an intervention was made at the individual hospital level. Demographic characteristics of infants and mothers comparing those born at BFHI and those not born at BFHI were examined using Chi squares tests.

Logistic and multinomial regression models were used to estimate the association of BFHI on ED visits and readmissions pre and post implementation in the three BFHI designated hospitals. These analyses were performed only on the patients born at the three BFHI hospitals. Exposure to the BFHI was determined by date of birth. Patients born at hospitals that did not adopt the BFHI were not included in these analyses so that readmissions and ED visits could be compared based on the temporal initiation of BFHI within the same hospitals, thus limiting confounding. Overall, four models were created in this series of analysis using the following dependent variables: (1) 30-day readmission, (2) 1-year readmissions (0, 1, 2 or more), (3) 30-day ED visit, (4) 1-year ED visits (0, 1, 2 or more). In all models the following independent variables were entered: (1) BFHI (born before or after initiation), (2) race, (3) preterm birth.

Two additional negative binomial regression models were fit to determine the effect of BFHI in babies born in the three BFHI hospitals. In these models the dependent variables were 1)number ofall cause one year ED visits and 2) number of all cause one year readmissions. Independent variables in these models included (1) BFHI (born before or after initiation), (2) race, (3) preterm birth.

We performed further analyses on the entire cohort, including those hospitals that did not participate in BFHI. In this series of analyses, patients born at the non-BFHI were included and served as controls. Interrupted Time Series Segmented Regression analysis was used to assess the effect the BFHI on the aggregate count of total ED visits and total readmission in all five hospitals - three BFHI designated hospitals and two control hospitals; 2016 was used as the interruption in the time series because all three BFHI designations occurred close to this time point. Interrupted time series can be used to study population-based intervention and segmented regression to study an outcome of interest. Interrupted time series also has the strength of being unaffected by some confounders that are likely to remain constant including population level socioeconomic status [10]. Analyses were conducted by using SAS version 9.4 (SAS Institute, Inc, Cary, NC) and R Core Team (2020).

## Results

The study sample included 19,695 babies with Medicaid insurance born at five hospital systems in Delaware. This included 15,939 (79.8%) that were born at three hospital systems that were part of the BFHI and 3,756 (20.2%) that were born in two hospital systems that did not participate in the BFHI. The demographics of the study sample are shown in Table 1. Babies born at BFHI hospitals were more likely to be Black race and less likely to be born prematurely than those born at non-BFHI- hospitals.

**Table 1:** Study demographics

Baby Characteristics	Overall Sample (n = 19,695)	BFHI (n = 15,939)	Non-BFHI (n = 3,756)
Race, n (%)			
Black	8111 (41.2)	6849 (42.9)	1262 (33.6)
white	10,820 (54.9)	8467 (53.1)	2353 (62.7)
Other	758 (3.9)	619 (3.9)	139 (3.7)
Male sex, n (%)	10,002 (50.8)	8112 (50.9)	1890 (50.3)
Preterm Birth, n (%)	1185 (6.0)	930 (5.8)	255 (6.8)

### Breast feeding related ED utilization and readmissions

ED utilization and readmissions over the first year of life for breastfeeding related diagnoses at the BFHI hospitals occurred in 240 (1.5%) and 226 (1.4%) of infants, respectively. These low rates of utilization precluded further analysis of specific breast-feeding related ED visits and readmissions by BFHI stratification.

### All cause 30-day and one year ED utilization and readmissions

#### Readmissions

Following initiation of BFHI at Delaware hospitals, there was an approximate 15% increased odds of all cause readmission in the 30 days following hospital discharge after adjusting for race and preterm birth, adjusted odds ratio 1.15, 95%CI 1.03–1.28 (Table 2). There were no differences in the odds of one, or two or more one-year all cause readmissions after BFHI (Table 2). Negative binomial regression models confirmed that one-year readmissions were unchanged after initiation of BFHI (incident rate ratio 1.12, 95% CI 0.99–1.26).

**Table 2:** Readmission and ED visits comparing pre and post BFHI using Logistic Regression. Analysis includes only the patients born at the hospital with BFHI certification

Outcomes	Pre-Baby Friendly Policy (n = 5514)	Post-Baby Friendly Policy (n = 10,425)	Baby Friendly Policy AOR 95% CI*
30-day Readmission (n, %)	660 (12.0)	1279 (12.3)	1.15 (1.03–1.28)
30-day ED visit (n, %)	498 (9.0)	856 (8.2)	0.90 (0.80–1.01)
One-Year Readmissions (n, %)			
0 Readmissions	4429 (80.4)	8502 (81.5)	Ref
1 Readmission	796 (14.4)	1365 (13.1)	0.94 (0.85–1.04)
2 or more	289 (5.2)	558 (5.4)	1.10 (0.95–1.28)
One-Year ED Visits (n, %)			
0 Visits	2526 (45.8)	5050 (48.5)	Ref
1 Visit	1327 (24.1)	2515 (24.1)	0.93 (0.86–1.02)
2 or more	1661 (30.1)	2860 (27.4)	0.85 (0.79–0.92)

*Models controlled for race and preterm birth

#### ED visits

There were no differences in all cause 30-day ED visits in BFHI hospitals after adjusting for race and preterm birth, adjusted odds ratio 0.90, 95%CI 0.80–1.01 (Table 2). There were no differences in patients receiving one ED visit pre and post BFHI. There was a reduced odds of babies requiring two or more ED Visits after BFHI (Table 2). Negative binomial regression confirmed that one-year ED visits were reduced (incident rate ratio 0.91, 95% CI 0.86–0.97) after controlling for race and preterm birth.

### Time series analysis of one year ED utilization and readmissions

**Fig. 1:**
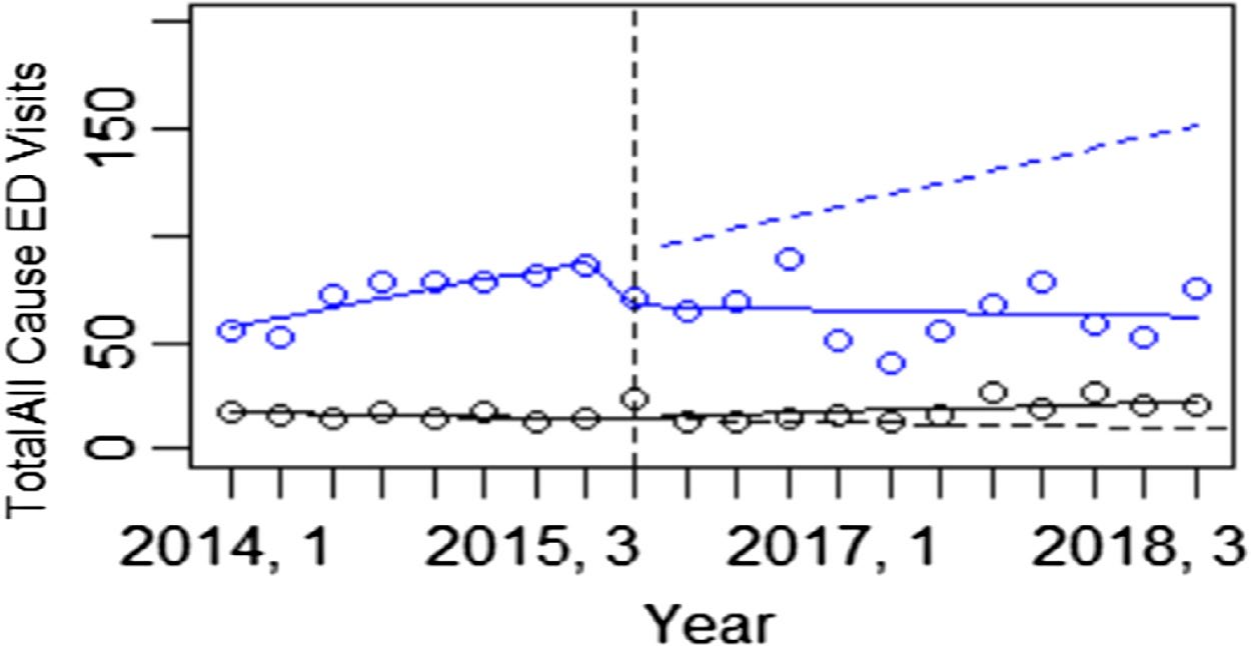
Interrupted time series: BFHI Hospitals vs. Control Hospitals ( One-Year ED Visit). Level change p = 0.07, Trend change p = < 0.01. Blue dots and lines represent BFHI hospital births while black dots and lines represent non-BFHI births. Each time point represents number of ED visits by quarter

**Fig. 2:**
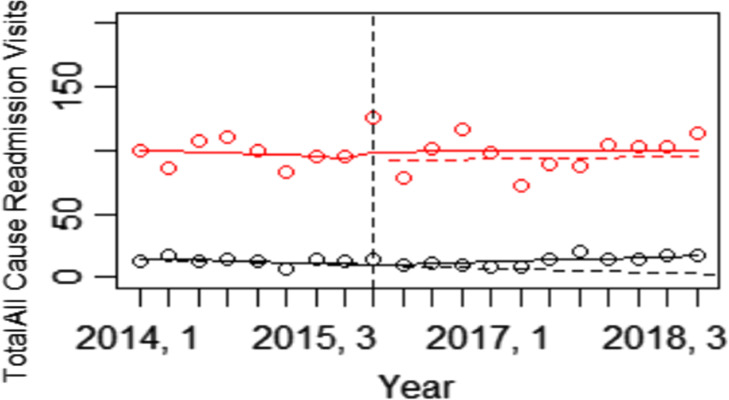
Interrupted time series: BFHI Hospitals vs. Control Hospitals (One-Year Readmissions). Level change p = 0.66, trend change p = 0.90 Red dots and lines represent BFHI hospital births while black dots and lines represent non-BFHI births. Each time point represents number of readmission by quarter

One-year readmissions and ED visits were compared in the babies born in the three hospitals systems that initiated BFHI to the two hospital systems that did not adopt the BFHI. Interrupted time series analysis showed that all cause ED visits were reduced in BFHI hospitals compared to non BFHI hospitals, β -5.90, p < 0.01 (Fig. 1) while readmission did not differ in the BFHI and non-BFHI hospital systems, β -0.34, p = 0.90 (Fig. 2).

## Discussion

The main findings of our study were that, in a statewide population of babies with Medicaid insurance, initiation of BFHI was associated with a small increase in all-cause 30-day readmission but no change in the odds of all cause one-year readmissions. There were no changes in 30-day all cause ED visits but one-year all cause ED visits were reduced following BFHI. Readmissions and ED visits directly related to breast feeding were uncommon in the study population.

To our knowledge, our study is unique in looking at the association of BFHI with readmission and ED utilization at the state level in a Medicaid population. While one study has shown that BFHI at a state level does not improve exclusive breast feeding at 3- and 6-months [7] other studies have shown BFHI to improve breast feeding rates [5, 11, 12]. Concern has also been raised about some of the specific components of BFHI including the provision of skin to skin care and risk of sudden unexpected postnatal collapse [13] and promotion of exclusive breast feeding has been suggested to potentially adversely impact maternal mental health [14]. In one mixed-method systemic review, BFHI was shown to promote unrealistic expectations on breast feeding and lack of understanding of formula feeding [15] which may increase readmission and ED utilization following initial hospitalization. We investigated both short term, 30-day, and one-year utilization to best determine the potential risks and benefits of BFHI. Readmission and ED visits in BFHI hospitals that were attributed to breast feeding were infrequent, occurring in 1.4% and 1.5% of the population respectively inclusive of the periods before and after BFHI initiation. One study showed that babies who are exclusively breast fed have a rate of readmission of 4%, almost double of formula fed infants [16]. It is difficult to compare our low rates of readmission to other populations as our readmission rates associated with potential breast-feeding related diagnoses were evaluated at a population level and not just in exclusively breast-feeding infants. This was done to investigate the BFHI as an intervention and explore some of the concerns that have been raised about BFHI, rather than the individual risks and benefits of breast feeding. Our analysis showed a modest increase in all-cause 30-day readmissions following BFHI. Hudson and colleagues have previously shown a reduction in hyperbilirubinemia without any change in readmission for hyperbilirubinemia following BFHI in a single center [17]. A study from a single center in the West Indies showed an increase in admission for hypernatremic dehydration following BFHI [18]. There are a few potential explanations for the small increase in 30-day readmissions following BFHI in our study sample. Although readmission associated with specific diagnoses related to breast feeding were uncommon, it is possible that the administrative data used in our study did not capture some diagnoses associated with breast feeding failure. The increase may have also resulted from unmeasured confounding associated with hospitals or community systems of care associated with the BFHI. Confirmation of an increase in all cause 30-day readmissions following BFHI in other populations is needed before this concern is considered by hospital systems deciding on initiating, continuing or discontinuing BFHI.

In contrast to the short-term increase in readmission associated with BFHI, our data showed a reduction in ED utilization over the first year following initial hospital discharge. To our knowledge, this study is the first to report an association of BFHI with a reduction in one year ED utilization after the initial hospital discharge. The observed reduction in ED utilization may have resulted from some of the known beneficial health effects of breast feeding including the reduction in respiratory related and other infections. We cannot rule out that other temporal factors including other unmeasured changes in statewide health care delivery, including improvements in primary care, could have led to the observed reduction. The findings from our study are important in evaluating the risks and benefits of adopting the BFHI. The low rate of breast-feeding related readmissions and ED utilization are reassuring, as is the potential longer-term reduction in ED utilization that was observed in our population. To provide better context of our findings, between 132 and 348 total babies in our study sample, had a reduction in two or more ED visits in the one year after birth associated with BFHI. On the other hand, between 20 and 184 babies in our study sample had a 30-day readmission associated with BFHI implementation in Delaware. The observed increase in all cause 30-day readmissions raises some concerns about the potential risks of BFHI and indicates the need to continuously monitor outcomes and mitigate any factors that may contribute to readmissions following birth. As our data are from a single state, further research on BFHI is necessary to validate and determine the generalizability of our findings.

Our study has several important strengths including the evaluation of BFHI at a state level and the use of Medicaid data to best allow ascertainment of healthcare utilization that crosses institutional boundaries. Our analysis is limited by not being able to show causality between BFHI and the observed outcomes. There was also likely unmeasured confounding that could have contributed to the observed results including variables such as maternal ethnicity, maternal body mass index, maternal diabetes and mode of delivery that were not available in our data set. Based on the source of our data we were not able to measure breast feeding at the individual level. Our data therefore best reflect system level changes on a population of newborns and not patient level decisions on breast feeding. Importantly, from our data we cannot determine if there were any changes in initiation or exclusivity of breast feeding based on BFHI and/or if any changes in these important feeding metrics influenced our observed outcomes. The choice to obtain BFHI designation is voluntary and thus the observed results may be reflective of many other hospital or multiple community level factors associated with the systems that chose to obtain certification. Our study sample only included patients with Medicaid insurance and thus may not be generalizable to other populations including commercial insurance. In addition, because we studied statewide healthcare utilization our data may not be generalizable for outcomes attributed to any one single center.

In summary, our study is the first to describe readmissions and ED utilization following BFHI at a statewide level. Our data show a small increase in 30 day all cause readmissions after BFHI balanced by a reduction in all cause ED utilization over the first year of life. Importantly, our findings must be interpreted with caution in the absence of knowing whether rates of breast feeding changed in babies with Medicaid insurance in Delaware associated with three hospitals starting the BFHI. Our study adds to the literature on the benefits and risks of BFHI and potentially helps to inform policy and decision making for statewide systems of care.

## Data Availability

All data were provided by the University of Delaware Center for Community Research and Service who hold an agreement with the Delaware Division of Medicaid and Medical Assistance. A de-identified data set was provided to ChristianaCare under a Delaware INBRE Core access award for the current study. Data are not publicly available.

## List of abbreviations

BFHI: Baby Friendly Hospital Initiative
